# Whole genome shotgun sequences of Streptococcus pyogenes causing acute pharyngitis from India

**DOI:** 10.1016/j.dib.2018.03.129

**Published:** 2018-04-01

**Authors:** Dhanya Dharmapalan, Francis Yesurajan Inbanathan, Suhas Kharche, Asmita Patil, Shrikrishna Joshi, Vijay Yewale, Jones Lionel Kumar Daniel, Kamini Walia, Balaji Veeraraghavan

**Affiliations:** a Apollo Hospitals, Navi Mumbai, Maharashtra, India; b Department of Clinical Microbiology, Christian Medical College, Vellore, Tamil Nadu, India; c Dr. Yewale Multispeciality Hospital for Children, Navi Mumbai, Maharashtra, India; d Joshi's Laboratory, Navi Mumbai, Maharashtra, India; e Indian Council of Medical Research, Ansari Nagar, New Delhi, India

## Abstract

*Streptococcus pyogenes*, belonging to group A streptococcus (GAS), causes over 600 million infections annually being a predominant human pathogen. Lack of genomic data on GAS from India is one limitation to understand its virulence and antimicrobial resistance determinants. The genome of GAS isolates from clinical samples collected at Navi Mumbai, India was sequenced and annotated. Sequencing was performed on Ion Torrent PGM platform. The size of annotated *S. pyogenes* genomes ranged from ~1.69 to ~1.85 Mb with coverage of 38× to 189×. Most of the isolates had *msr(D)* and *mef(A)*, and four isolates had *erm(B)* gene for macrolide resistance. The genome harboured multiple virulence factors including exotoxins in addition to phage elements in all GAS genomes. Four isolates belonged to sequence type ST28, 7 were identified as ST36 and 1 as ST55.

**Specifications table**

**Table:** 

Subject area	*Biology*
More specific subject area	*Microbial genome*
Type of data	*Whole genome shotgun sequences*
How data was acquired	*Ion Torrent PGM*
Data format	*Analyzed genome sequence*
Experimental factors	S. pyogenes *strains were cultured on blood agar medium. Genomic DNA from cultures were isolated using QIAamp DNA mini kit (Qiagen, Germany).*
Experimental features	*Sequencing was performed according to Ion Torrent PGM specific protocols for library preparation and DNA-seq*.
Data source location	*Mumbai, India, 19.0760°N, 72.8777°E*
Data accessibility	*Genome data are available at GenBank under the accession numbers*
	*NGQI00000000, NGQK00000000, NGQL00000000, NGQN00000000, NIYX00000000, NJPV00000000, NIYZ00000000, NGQM00000000, NGQJ00000000, NGQO00000000, NGQP00000000, NIYY00000000*
	https://www.ncbi.nlm.nih.gov/nuccore/NGQI00000000
	https://www.ncbi.nlm.nih.gov/nuccore/NGQK00000000
	https://www.ncbi.nlm.nih.gov/nuccore/NGQL00000000
	https://www.ncbi.nlm.nih.gov/nuccore/NGQN00000000
	https://www.ncbi.nlm.nih.gov/nuccore/NIYX00000000
	https://www.ncbi.nlm.nih.gov/nuccore/NJPV00000000
	https://www.ncbi.nlm.nih.gov/nuccore/NIYZ00000000
	https://www.ncbi.nlm.nih.gov/nuccore/NGQM00000000
	https://www.ncbi.nlm.nih.gov/nuccore/NGQJ00000000
	https://www.ncbi.nlm.nih.gov/nuccore/NGQO00000000
	https://www.ncbi.nlm.nih.gov/nuccore/NGQP00000000
	https://www.ncbi.nlm.nih.gov/nuccore/NIYY00000000

**Value of the data**

• Group A streptococcus (GAS) causes over 600 million throat infections annually being a predominant human pathogen with high genomic plasticity due to the prophage integration and horizontal gene transfer.
• This is the first genome report of *S. pyogenes* from India available in public database.
• The GAS genomic data will serve as a base for further research focusing on the genomic attributes of virulence, antimicrobial resistance and clonal association by Whole genome shotgun sequencing.

## 1 Data

*Streptococcus pyogenes*, belonging to group A streptococcus (GAS), causes over 600 million infections annually being a predominant human pathogen. GAS throat infections are common in children between 4 and 7 years and pose several clinical and public health challenges [1]. Prevalence of Pharyngitis caused by *S. pyogenes* is difficult to determine as it is a throat colonizer, but some studies report as 10–15% [2]. The GAS pharyngitis is usually undetermined due to its self-limiting nature and major cases being of viral etiology [3]. M proteins, pili, leukocidins, streptolysins (O,S), complement inhibiting proteins, immunoglobulin-degrading enzymes, and superantigens are genome-encoded virulence factors that have been well characterized in *S. pyogenes*, [4], [5], where efflux pumps and leukocyte evasion strategies stays as an integral factors. High genomic plasticity is seen in *S. pyogenes* due to the prophage integration and horizontal gene transfer. [6].

The post Streptococcal sequelae following GAS pharyngitis are the non-suppurative manifestation of rheumatic fever followed by Rheumatic heart disease. In India, the overall prevalence is estimated at 1.5–2/1000 in all age groups, (total population about 1.3 billion) being suggestive of 2.0 to 2.5 million patients of RHD in the country [4]. Due to the high burden of the GAS infections in India, preventive strategies like vaccination turn to be the need of the hour.

Furthermore, lack of genomic data on GAS from India is one limitation to understand its virulence and antimicrobial resistance determinants. This study reports the whole genome sequence data of *S. pyogenes* for the first time from India. The GAS genomic data will serve as a base for further research focusing on the genomic attributes of virulence, antimicrobial resistance and clonal association by Whole genome shotgun sequencing.

## 2 Experimental design, materials and methods

### 2.1 Study isolates

During the months of March–May 2017, children up to 18 years with acute pharyngitis were screened for GAS infections at Dr. Yewale Multispeciality Hospital for Children, Navi Mumbai using the cutoff score of 3 of the Modified Centor criteria.

### 2.2 DNA extraction and genome sequencing

A total of 12 culture confirmed *S. pyogenes* were subjected to total DNA extraction using QiAamp DNA mini Kit (Qiagen, Germany).Whole genome shotgun sequencing was performed using IonTorrent PGM platform (Life Technologies) with 400 bp chemistry.

### 2.3 De novo assembly and annotation

Assembly of the raw reads were performed using AssemblerSPAdes v.5.0.0.0 embedded in Torrent suite server v.5.0.5. Annotation of the genome were done using the PATRIC database (the bacterial bioinformatics database and analysis resource) (http://www.patricbrc.org), [7] and the NCBI Prokaryotic Genome Automatic Annotation Pipeline (PGAAP) (http://www.ncbi.nlm.nih.gov/genomes/static/Pipeline.html). Further genome analysis was performed with the genomic tools available at the Center for Genomic Epidemiology (CGE) server (http://www.cbs.dtu.dk/services), and PATRIC database. The size of annotated *S. pyogenes* genomes ranged from ~1.69 to ~1.85 Mb with coverage of 38X to 189X (Table 1). The number of Coding DNA sequences (CDS) per genome ranged between 1725 and 2042. The draft genome sequences have been deposited in DDBJ/ENA/GenBank under the accession numbers provided in Table 1. The version described in this manuscript is version 1.

**Table 1:** Clinical and genome data of Group A Streptococcus (*n*=12).

**Isolate ID**	**Age in years/Gender**	**Resistance**			**Fever defervescence**	**Compliance to total duration antibiotic**	**Recurrence**	**Sequence Types**	**emm Type**	**Total size (bp)**	**Coverage**	**CDS**	**Contigs**	**AMR genes**	**Plasmids**	**Accession**
		**Penicillin/Amoxicillin**	**Clindamycin**	**Macrolide**												
MUMCMC2276	7.6/F	No	Yes	Yes	2	Yes	Yes	36	*emm*12.0 (*emm*-cluster A-C4)	1727473	184	1754	50	*msr(D), mef(A)*	–	NGQI00000000
MUMCMC661	6.5/M	No	No	Yes	NA	NA	NA	36	*emm*12.4 (*emm*-cluster A-C4)	1852181	174	1967	62	*msr(D), mef(A)*	–	NGQK00000000
MUMCMC650	2.4/F	No	Yes	Yes	NA	NA	NA	36	*emm*12.0 (*emm*-cluster A-C4)	1691843	164	1725	49	–	–	NGQL00000000
MUMCMC317	5/F	No	No	Yes	4	Yes	No	36	*emm*12.0 (*emm*-cluster A-C4)	1750987	189	1776	62	*msr(D), mef(A)*	–	NGQN00000000
MUMCMC1953	3.5/F	No	No	No	2	Yes	No	36	*emm*12.0 (*emm*-cluster A-C4)	1840495	115	1886	49	*msr(D), mef(A)*	–	NIYX00000000
MUMCMC2034	2.5/M	No	Yes	No	4	Yes	No	36	*emm*12.0 (*emm*-cluster A-C4)	1747918	136	1762	43	*msr(D), mef(A)*	–	NJPV00000000
MUMCMC261	2/M	No	No	No	2	Yes	No	36	*emm*12.0 (*emm*-cluster A-C4)	1732451	129	1752	53	*msr(D), mef(A)*	–	NIYZ00000000
MUMCMC616	6/M	No	No	Yes	2	Yes	No	28	*emm*1.0. (*emm* cluster A-C3)	1856054	38	2042	66	*aph(3')-III, ant(6)-Ia, erm(B), tet(M)*	–	NGQM00000000
MUMCMC662	5/M	No	No	No	1	Yes	No	28	*emm*1.0. (*emm* cluster A-C3)	1849506	88	1966	38	*aph(3')-III, ant(6)-Ia, erm(B), tet(M)*	–	NGQJ00000000
MUMCMC51	5/M	No	No	Yes	1	Yes	No	28	*emm*1.0. (*emm* cluster A-C3)	1849373	134	1912	39	*aph(3')-III, ant(6)-Ia, erm(B), tet(M)*	–	NGQO00000000
MUMCMC13	6/F	No	No	Yes	2	Partial (7 days)	Yes	28	*emm*1.0. (*emm* cluster A-C3)	1852166	169	1917	51	*aph(3')-III, ant(6)-Ia, erm(B), tet(M)*	–	NGQP00000000
MUMCMC433	5.5/F	No	No	No	2	No antibiotic prescribed	No	55	*emm*2.0 (*emm*-cluster E4)	1863902	121	1921	33	*msr(D), mef(A)*	–	NIYY00000000

*NA- not available (patient couldn’t be followed).

Antimicrobial resistance (AMR) genes and plasmids were screened with ResFinder 2.1 and PlasmidFinder 1.3 tools [8], [9]. Most of the isolates had *msr(D)* and *mef(A)*, and four isolates had *erm(B)* gene for macrolide resistance. Isolates MUMCMC616, MUMCMC662, MUMCMC51 and MUMCMC13 had *aph(3')-III*, *ant(6)-Ia*, and *tet*(M) genes for aminoglycoside and tetracycline resistance respectively (Table 1). Also, PATRIC analysis revealed ABC transporter membrane-spanning permease, multidrug resistance efflux pump *pmrA* and multi antimicrobial extrusion (MATE) family transporter genes responsible for macrolide and multi-drug resistance in all isolates.

Multiple virulence determinants in the GAS genomes were identified using the annotated data from PATRIC (Table 2). Of which, all the genomes harboured streptolysins O & S, and Streptococcal pyrogenic exotoxins C and G. Clusters of regularly interspaced short palindromic repeats (CRISPR) and spacer sequences in the genome were identified using CRISPR finder (http://crispr.u-psud.fr/Server/) [10]. All isolates carried 1,2,3,4,5d CRISPR type with varied repeat, spacer and array regions (Table 3).

**Table 2:** Description of the presence virulence traits in GAS genomes of the study.

Virulence trait	MUMCMC2276	MUMCMC661	MUMCMC650	MUMCMC317	MUMCMC1953	MUMCMC2034	MUMCMC261	MUMCMC616	MUMCMC662	MUMCMC51	MUMCMC13	MUMCMC433	**Gene(s) with potential for conferring virulence traits**
Antiphagocytic M protein	+	+	+	+	+	+	+	+	+	+	+	+	*emm*, *enn*X, *fbp*, *iga*R
Streptokinase	+	+	+	+	+	+	+	+	+	+	+	+	ska
CAMP factor	+	+	+	+	+	+	+	+	+	+	+	+	*cfa*
Streptolysin O	+	+	+	+	+	+	+	+	+	+	+	+	*slo*
Streptolysin S	+	+	+	+	+	+	+	+	+	+	+	+	*sag*B, C, D, E, F, H, I, asn-ORF, ABC transporter
Putative peptidoglycan hydrolase	+	+	+	+	+	+	+	+	+	+	+	+	GbpB/SagA/PcsB
Hyaluronate lyase precursor	+	+	+	+	+	+	+	+	+	+	+	+	*hyl*
Hyaluronan synthase	–	+	+	+	+	+	+	+	+	+	+	+	*has*A
Exotoxin*	+	+	+	+	+	+	+	–	–	–	–	–	Scarlet fever
Streptococcal pyrogenic exotoxin A*	–	–	–	–	+	–	–	+	+	+	+	–	*spe*A
Cysteine Protease B*	+	+	+	+	+	+	+	+	+	+	+	+	speB
Streptococcal pyrogenic exotoxin C*	+	+	+	+	+	+	+	+	+	+	+	+	speC
Streptococcal pyrogenic exotoxin G	+	+	+	+	+	+	+	+	+	+	+	+	speG
Streptococcal pyrogenic exotoxin H*	–	+	–	–	+	–	–	–	–	–	–	–	speH
Streptococcal pyrogenic exotoxin I*	–	_+_	–	–	–	–	–	–	–	–	–	–	speI
Streptococcal pyrogenic exotoxin J	–	–	–	–	+	–	+	+	+	+	+	–	speJ
Streptococcal pyrogenic exotoxin K*	–	–	–	–	–	–	–	–	–	–	–	+	speK
Streptococcal pyrogenic exotoxin L*	–	–	–	–	–	–	–	–	–	–	–	–	speL
Streptococcal pyrogenic exotoxin M*	–	–	–	–	–	–	–	–	–	–	–	–	speM
Streptococcal mitogenic exotoxin Z	+	+	+	+	+	+	+	+	+	+	+	–	smeZ
C5a peptidase	+	+	+	+	+	+	+	+	+	+	+	+	scpA
Secreted endo-beta-Nacetylglucosaminidase	+	+	+	+	+	+	+	+	+	+	+	+	ndoS
Streptococcal inhibitor of complement	_	–	–	–	–	–	–	+	+	+	+	+	*sic*
Exotoxin nucleases	–	–	–	–	–	–	–	–	–	–	–	–	*spd*1, 2, 3, 4, *sda*
Immunoglobulin-binding protease	+	+	+	+	+	+	+	+	+	+	+	+	*ide*S
Collagen-like surface proteins	+	+	+	+	+	+	+	+	+	+	+	+	*scl*A, B

**Table 3:** Details of the of CRISPR/CAS types and occurrence in GAS genomes.

Isolate	CRISPR/CAS type	CRISPR Repeat	CRISPR Spacer	CRISPR array
MUMCMC2276	1,2,3,4,5d	9	7	2
MUMCMC662	1,2,3,4,5d	9	7	2
MUMCMC661	1,2,3,4,5d	4	3	1
MUMCMC650	1,2,3,4,5d	9	7	2
MUMCMC616	1,2,3,4,5d	9	7	2
MUMCMC317	1,2,3,4,5d	9	7	2
MUMCMC51	1,2,3,4,5d	9	7	2
MUMCMC13	1,2,3,4,5d	9	7	2
MUMCMC1953	1,2,3,4,5d	9	7	2
MUMCMC433	1,2,3,4,5d	7	5	2
MUMCMC2034	1,2,3,4,5d	9	7	2
MUMCMC261	1,2,3,4,5d	9	7	2

Multi-locus sequence typing (MLST) of the GAS isolates were interpreted with the standard references available at the MLST 1.8 database (https://cge.cbs.dtu.dk//services/MLST/). Four isolates belonged to ST28, 7 were identified as ST36 and 1 as ST55. M protein typing was done using the Blast 2.0 server provided by National Centers for Disease Control, Biotechnology Core Facility Computing Laboratory and *emm* types were assigned. Isolates with ST28 corresponds to *emm*1.0 (*emm* cluster A-C3), ST36 to *emm*12.0 (*emm*-cluster A-C4) and ST55 to *emm*2.0 (*emm*-cluster E4) (Table 1).

The phages and phage associated elements in the genome of GAS were identified using PHAge Search Tool Enhanced Release (PHASTER) [11] (Table 4). Strept 315.2 phage was associated to all ST36 isolates with Clostr phiCT453B, Strept P9, Strept phiARI0131, Lactoc_PLgT, Strept phiARI0462, were the other phages seen. ST28 harboured PHAGE_Strept_T12, PHAGE Lactoc 28201, PHAGE Strept 315.3, PHAGE Pseudo phi3, PHAGE Strept 315.2 and PHAGE Strept T12 consistently among all isolates. PHAGE Strept 315.4, PHAGE Strept T12 and Clostr_phiCT453B were seen in ST55 isolate.

**Table 4:** Identity of putative phages and phage elements detected in GAS genomes.

Isolate	Phage Name	Size	GC %	CDS
MUMCMC2276	PHAGE_Strept_315.2_NC_004585	24.3Kb	37.65	15
	PHAGE_Clostr_phiCT453B_NC_029004	49.8Kb	39.51	47
MUMCMC661	PHAGE_Strept_315.2_NC_004585	38Kb	37.69	47
	PHAGE_Lactoc_PLgT_1_NC_031016	63.1Kb	39.14	66
	PHAGE_Strept_P9_NC_009819	33.2Kb	39.73	42
	PHAGE_Strept_phiARI0131_2_NC_031941	26.2Kb	38.91	36
MUMCMC650	PHAGE_Strept_315.2_NC_004585	21.7Kb	36.94	16
MUMCMC317	PHAGE_Clostr_phiCT453A_NC_028991	39.2Kb	40.66	45
	PHAGE_Strept_315.2_NC_004585	21.2Kb	37.01	16
	PHAGE_Strept_P9_NC_009819	16Kb	39.11	24
MUMCMC1953	PHAGE_Strept_phiARI0462_NC_031942(6)	25.1Kb	37.29	25
	PHAGE_Clostr_phiCT453A_NC_028991(12)	39.2Kb	40.66	45
	PHAGE_Strept_P9_NC_009819(30)	32.6Kb	39.84	41
	PHAGE_Strept_phiARI0131_2_NC_031941(8)	29Kb	38.73	40
	PHAGE_Strept_315.2_NC_004585(17)	11.7Kb	37.58	21
MUMCMC2034	PHAGE_Clostr_phiCT453A_NC_028991(12)	39.2Kb	40.66	45
	PHAGE_Strept_315.2_NC_004585(7)	21Kb	36.96	16
MUMCMC261	PHAGE_Clostr_phiCT453A_NC_028991(12)	39.2Kb	40.66	45
	PHAGE_Strept_315.2_NC_004585(7)	21Kb	36.96	16
MUMCMC616	PHAGE_Strept_T12	28.2Kb	38.55	45
	PHAGE_Lactoc_28201_NC_031013	21.8Kb	37.58	25
	PHAGE_Strept_315.3_NC_004586	15.9Kb	36.07	31
	PHAGE_Pseudo_phi3_NC_030940	20.7Kb	35.75	26
	PHAGE_Strept_315.3_NC_004586	20.9Kb	38.56	35
	PHAGE_Strept_T12_NC_028700	20Kb	35.94	29
	PHAGE_Strept_315.2_NC_004585	21.1Kb	39.64	25
MUMCMC662	PHAGE_Strept_T12_NC_028700	28.2Kb	38.55	46
	PHAGE_Lactoc_28201_NC_031013	30Kb	37.60	27
	PHAGE_Strept_315.2_NC_004585	21.1Kb	39.64	26
	PHAGE_Strept_315.3_NC_004586	15.8Kb	36.07	32
	PHAGE_Pseudo_phi3_NC_030940	20.7Kb	35.76	26
	PHAGE_Strept_315.3_NC_004586	20.9Kb	38.58	32
	PHAGE_Strept_T12_NC_028700	20Kb	35.94	29
MUMCMC51	PHAGE_Strept_315.2_NC_004585	20.9Kb	39.68	27
	PHAGE_Strept_T12_NC_028700	28.4Kb	38.54	43
	PHAGE_Lactoc_28201_NC_031013	30Kb	37.60	26
	PHAGE_Strept_315.3_NC_004586	15.6Kb	36.09	31
	PHAGE_Pseudo_phi3_NC_030940	20.6Kb	35.77	26
	PHAGE_Strept_315.3_NC_004586	20.7Kb	38.61	31
	PHAGE_Strept_T12_NC_028700	19.7Kb	35.97	27
MUMCMC13	PHAGE_Strept_T12_NC_028700	28.1Kb	38.56	43
	PHAGE_Lactoc_28201_NC_031013	30Kb	37.60	26
	PHAGE_Pseudo_phi3_NC_030940	22.1Kb	35.81	26
	PHAGE_Strept_315.3_NC_004586	15.9Kb	36.07	32
	PHAGE_Strept_315.3_NC_004586	20.8Kb	38.58	33
	PHAGE_Strept_T12_NC_028700	20Kb	35.95	28
	PHAGE_Strept_315.2_NC_004585	21Kb	39.64	26
MUMCMC433	PHAGE_Strept_T12_NC_028700(23)	22.4Kb	38.89	34
	PHAGE_Clostr_phiCT453B_NC_029004(11)	49.8Kb	39.51	47
	PHAGE_Strept_315.4_NC_004587(17)	22.3Kb	37.81	21
